# EphB4 forward signalling mediates angiogenesis caused by *CCM3/PDCD10*‐ablation

**DOI:** 10.1111/jcmm.13105

**Published:** 2017-04-01

**Authors:** Chao You, Kai Zhao, Philipp Dammann, Kathy Keyvani, Ilonka Kreitschmann‐Andermahr, Ulrich Sure, Yuan Zhu

**Affiliations:** ^1^ Department of Neurosurgery University of Duisburg‐Essen Essen Germany; ^2^ Department of Neurosurgery Tongji Hospital Tongji Medical College Huazhong University of Science and Technology Wuhan China; ^3^ Institute of Neuropathology University of Duisburg‐Essen Essen Germany

**Keywords:** angiogenesis, EphB4 forward signalling, DLL4‐Notch signalling, CCM3/PDCD10

## Abstract

CCM3, also named as PDCD10, is a ubiquitous protein expressed in nearly all tissues and in various types of cells. It is essential for vascular development and post‐natal vessel maturation. Loss‐of‐function mutation of *CCM3* predisposes for the familial form of cerebral cavernous malformation (CCM). We have previously shown that knock‐down of *CCM3* stimulated endothelial angiogenesis via impairing DLL4‐Notch signalling; moreover, loss of endothelial CCM3 stimulated tumour angiogenesis and promoted tumour growth. The present study was designed to further elucidate the inside signalling pathway involved in *CCM3‐*ablation‐mediated angiogenesis. Here we report for the first time that silencing endothelial *CCM3* led to a significant up‐regulation of EphB4 mRNA and protein expression and to an increased kinase activity of EphB4, concomitantly accompanied by an activation of Erk1/2, which was reversed by treatment with the specific EphB4 kinase inhibitor NVP‐BHG712 (NVP), indicating that silencing *CCM3* activates EphB4 kinase forward signalling. Furthermore, treatment with NVP rescued the hyper‐angiogenic phenotype induced by knock‐down of endothelial *CCM3 in vitro* and *in vivo*. Additional study demonstrated that the activation of EphB4 forward signalling in endothelial cells under basal condition and after *CCM3*‐silence was modulated by DLL4/Notch signalling, relying EphB4 at downstream of DLL4/Notch signalling. We conclude that angiogenesis induced by *CCM3*‐silence is mediated by the activation of EphB4 forward signalling. The identified endothelial signalling pathway of CCM3‐DLL4/Notch‐EphB4‐Erk1/2 may provide an insight into mechanism of *CCM3‐*ablation‐mediated angiogenesis and could potentially contribute to novel therapeutic concepts for disrupting aberrant angiogenesis in CCM and in hyper‐vascularized tumours.

## Introduction

Cerebral cavernous malformation 3 (CCM3) is also named programmed cell death 10 (PDCD10). Loss‐of‐function mutation of *CCM3* gene causes cerebral cavernous malformation (CCM), one of the most common vascular disorders involving aberrant angiogenesis in the central nervous system 1. It is known that CCM3 can act in a protein complex of CCM1–CCM2–CCM3, thereby sharing common signalling pathways 2, 3. Apart from that, CCM3 also displays distinct functions. As a pleiotropic molecule, CCM3 is involved in angiogenesis, vessel permeability, apoptosis and senescence, oxidative metabolism and Golgi complex polarization 4 and is also essential for the neuron‐glial unit 5. We and others have identified a variety of signalling pathways such as p‐Akt, p38, p‐Erk, VEGFR2, STK24/25, MST4, RhoA, DLL4‐Notch and SMAD underlying the diverse functions of CCM3 6. More recently, loss of endothelial CCM3 has been shown to activate MEKK3‐KLF2/4 and rapamycin (mTOR) signalling pathways resulting in defects of vascular development 7 and defective autophagy 8, respectively.

The Eph receptors and their ephrin ligands comprise the largest subfamily of receptor tyrosine kinases (RTKs). EphB4 is mainly expressed in endothelial cells and is essential for endothelial function and angiogenesis 9. Activation of EphB4 by ephrinB2 promotes endothelial adhesion, cell proliferation, tube formation, migration and cytoskeletal organization, whereas inactivation of ephb4 in mice results in embryonic lethality due to arrested angiogenesis 10. Blockade of EphB4 and ephrinB2 activities by soluble EphB4 (sEphB4) suppresses angiogenesis mediated by VEGF and bFGF 11. Moreover, up‐regulation of EphB4 has been found in various types of solid tumours and is associated with hyper‐angiogenesis and with poor prognosis 12, 13.

DLL4‐Notch is another important signalling for regulating vascularisation, angiogenesis as well as post‐angiogenic vessel remodelling and vessel maturation 14. Increasing evidence emphasizes the crosstalk of DLL4‐Notch and ephrinB2/EphB4 signalling cascades in regulating endothelial cell function 15. Activation of Notch signalling and mutation in ephrinB2 and EphB4 both cause the same phenotype of arteriovenous malformations in mice 16. We have recently reported the implications of DLL4‐Notch and EphrinB2‐EphB4 signalling in human hyper‐vascularized brain tumours including glioblastoma (GBM) 17, hemangiopericytoma 18 and hemangioblastoma 19. In support of this, a recent study has shown that the combination of DLL4‐Notch and EphrinB2/EphB4 targeted therapy is highly effective in disrupting tumour angiogenesis 20.

We have previously demonstrated that CCM3 is deficient in the endothelial cells of the CCM lesion derived from *CCM3*‐mutation carriers and that loss of endothelial CCM3 stimulates angiogenesis via impairing DLL4‐Notch signalling 21, 22. More recently, we have discovered that CCM3/PDCD10 is absent in the majority of tumour vessels of GBM, the most common and aggressive brain tumour characterized by massive neo‐angiogenesis 23. Remarkably, knock‐down of endothelial CCM3/PDCD10 largely stimulated neo‐angiogenesis and promoted tumour growth through a paracrine mechanism 23. These findings indicate a crucial role of CCM3 not only in CCM, but also in tumours associated with aberrant angiogenesis.

The present study was designed to explore whether EphB4 forward signalling is involved in angiogenesis mediated by ablation of endothelial CCM3, and if so, how EphB4 and DLL4‐Notch signalling, which we have identified in our previous study to be targeted by *CCM3*‐silence 22, coordinate each other in angiogenesis resulting from CCM3‐ablation.

## Materials and methods

### Cell culture and treatment

Human umbilical vein endothelial cells (HUVEC) were cultured in endothelial cell growth medium (ECGM) with supplement (Promocell, Heidelberg, Germany). To study the signalling pathways, cells were treated with different inhibitors and activators including a specific EphB4 kinase inhibitor NVP‐BHG712 (NVP) (kind gift from Novartis, Basel, Switzerland), ephrinB2‐Fc (B2fc; R&D System, Wiesbaden, Germany), recombinant human DLL4 (rhDLL4; R&D System) and γ‐secretase inhibitor DAPT (Sigma‐Aldrich, Munich, Germany) as indicated in the individual experiments.

### Silencing *CCM3* by siRNA and by lentiviral transduction of shRNA

Silencing *CCM3* by siRNA transfection was carried out as described in previous studies 21, 22. The stable knock‐down of the *CCM3* gene by lentiviral transduction of shRNA was performed as described in our recent publication 23.

### Human endothelial spheroid‐based angiogenesis model in mice and the treatment

The animal experiments were performed strictly according to the approved ethics contract with the local government (Nr.: 84‐02.04.2012.A348). The spheroid‐based angiogenesis model was established according to Laib *et al*. 24 with modifications. We used female nude mice (4–6 weeks old, *n* = 4 for each group) which were implanted subcutaneously in their left flank with spheroids formed by *CCM3‐*knock‐down endothelial cells (shCCM3) or by empty vector‐transduced control cells (EV). The shCCM3 and EV‐transduced cells were prepared as described previously 23. To maintain the stable knock‐down of *CCM3 in vivo*, the animals received drinking water containing 2 mg/ml of doxycycline (Sigma‐Aldrich) from the 1st day. After 20 days, the plugs were removed from the mice immediately after cervical dislocation and were used for sectioning or for extraction of RNA and protein, respectively. NVP was treated to the mice (8 mg/kg, i.g.) every 2nd day beginning at the 1st day to the 18th day after the implantation of the spheroids. The control mice received vehicle only.

### RNA extraction, cDNA synthesis and real‐time PCR (RT^2^‐PCR)

Total RNA was extracted using the innuPREP RNA mini kit (Analytik jena, Berlin, Germany). The cDNA was synthesized using the iscript cDNA kit (Bio‐Rad, Munich, Germany). The PCR reaction mixture was prepared to a final volume of 15 μl comprising of 6 μl of cDNA template (4 ng/μl), 7.5 μl of SYBR green supermix (Bio‐Rad), 0.3 μl of forward and reverse specific primers (10 μM) and Rnase‐free H2O. Real‐time PCR was performed on an iQ5 PCR instrument by using three‐step programme parameters as follows: 15 min. at 95°C for denaturation and then 40 cycles of amplification at 95°C for 30 sec., annealing at 60°C for 30 sec. and 72°C for 50 sec., 95°C for 1 min. and 55–95°C with a heating rate of 0.5°C every 10 sec. Glyceraldehyde‐3‐phosphate dehydrogenase (GAPDH) was stably detected and was used as the reference gene. The relative expression of target gene was calculated by 2^−ΔΔCt^ method as described previously 22. Primer sequences for individual genes are listed in Table 1.

**Table 1:** Primer sequences for real‐time RT‐PCR

Primer	Sequence
*CCM3*	
for.	TGG CAG CTG ATG ATG TAG AAG
rev.	TCG TGC CTT TTC GTT TAG GT
*EphB4*	
for.	TGT GTT GGA GGG AAC CTG TTT C
rev.	GGG CCC CTG TTT CAA CTT G
*GAPDH*	
for.	AGC CAC ATC GCT CAG ACA
rev.	GCC CAA TAC GAC CAA ATC C

for. forward; rev. reverse.

### Western blotting

Total protein extraction, electrophoresis and blotting were performed according to a previous protocol 22. The blots were incubated at 4°C overnight with the following primary antibodies: rabbit anti‐CCM3 (Atlas Antibodies, Munich, Germany), rabbit anti‐EphB4 (Santa Cruz Technology, Heidelberg, Germany), rabbit anti‐DLL4, p‐Erk1/2, GAPDH and mouse anti‐p‐Akt (Cell Signaling, Frankfurt, Germany), mouse anti‐Hey1 (Abcam, Frankfurt, Germany) and rabbit anti‐actin (Sigma‐Aldrich). To semi‐quantify the blot, integrated optical density (IOD) of the bands was measured by using Image J software. The IOD ratio of the target protein to the housekeeping protein (Actin or GAPDH) was calculated, and the relative expression of the target protein was normalized as the percentage of the control.

### Immunofluorescent staining

Immunofluorescent staining was performed according to the protocol described previously 23. For EphB4 single staining, rabbit anti‐EphB4 antibody (1:400) (Santa Cruz Technology) was used. For double staining, the following antibody mixtures were applied to the sections: mouse anti‐CD31 (1:40; Dako, Hamburg, Germany) and rabbit anti‐PDCD10 (1:100; Atlas Antibodies, Stockholm, Sweden). Negative control sections were incubated with nonimmune IgG. Counterstaining was performed with Hoechst‐33258. The images were acquired using a fluorescence microscope (Axio Imager M2; Zeiss, Wetzler, Germany).

### Detection of kinase activity of EphB4 (p‐EphB4) by enzyme‐linked immunosorbent assay (ELISA)

The level of p‐EpB4, referring the kinase activity of EphB4, was detected by using an ELISA kit according to the manufacture's instruction (R&D Systems).

### Detection of proliferation, migration tube formation and sprouting

Cell proliferation, migration, tube formation and sprouting of endothelial cells were studied according to previously established protocols 22.

### Statistics

Statistical analysis was performed using WinSTAT. Data were presented as mean and standard deviation (mean ± SD). Results between two groups were analysed by Student's *t*‐test. Differences between multiple groups were analysed by using ANOVA followed by the Scheffé test. A *P* value < 0.05 was considered statistically significant.

## Results

### Up‐regulation of the expression and kinase activity of EphB4 in *CCM3*‐silenced endothelial cells

**Figure 1:**
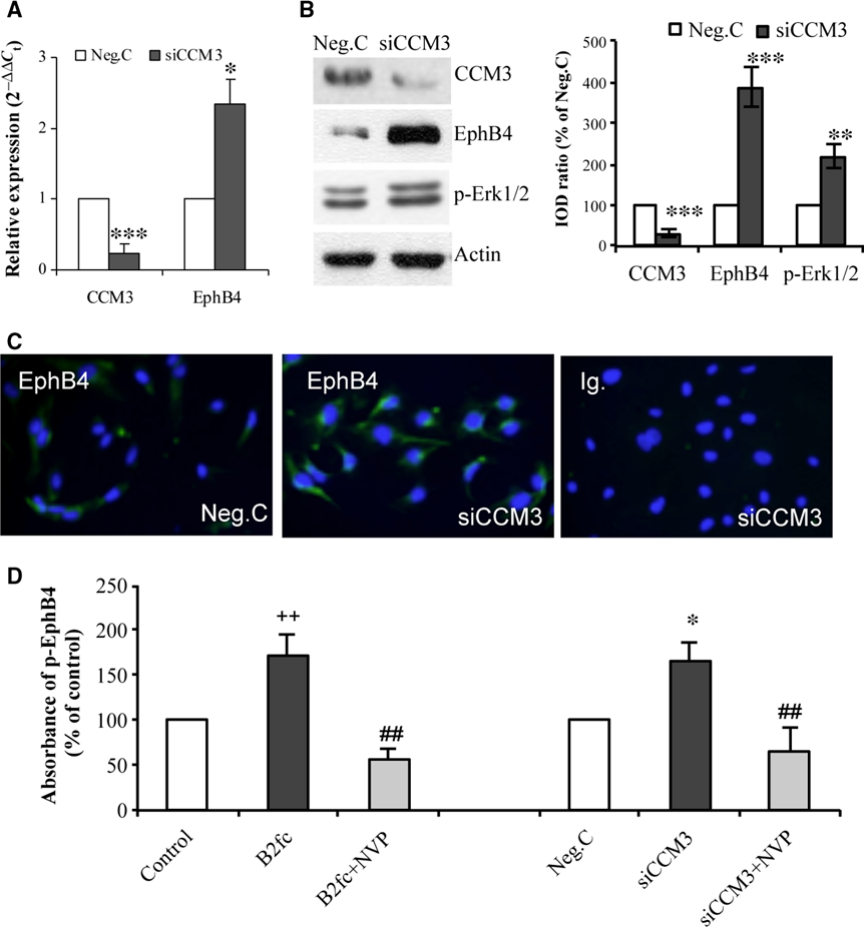
Silencing *CCM3* increased the expression and activity of EphB4 in endothelial cells. Cells were transfected either with 70 nM of specific siRNA targeting *CCM3* (siCCM3) or with a control siRNA (Neg.C). (**A**) Silencing *CCM3* up‐regulated mRNA level of EphB4. Total RNA was extracted 48 hrs after the transfection for real‐time RT‐PCR (RT ^2^‐PCR). (**B**) Silencing *CCM3* increased the EphB4 protein expression and activated Erk1/2. The total protein was harvested 72 hrs after the transfection for Western blot. The integrated optical density (IOD) ratio of the target protein to housekeeping protein actin was then calculated and normalized as the percentage of the control (Neg.C). (**C**) Silencing *CCM3* increased the immunoreactivity of EphB4. Ig.: negative control staining with unspecific immunoglobulin. (**D**) Silencing *CCM3* elevated the level of phospho‐EphB4 (p‐EphB4), which was abolished by a specific EphB4 inhibitor NVP‐BHG712 (NVP). NVP (10 nM) was treated to cells at 48 hrs after siCCM3‐transfection followed by the incubation for 90 min. As a positive control, cells received ephrinB2‐Fc (B2fc) (1 μg/ml) for 30 min. The level of p‐EphB4 was measured by ELISA. The absorbance was detected at 450 nm and normalized as the percentage of the control group. All data presented in A–D were representative of at least three independent experiments. *P < 0.05, **P < 0.001 and ***P < 0.001, compared with Neg.C; ^++^ *P* < 0.01, compared with control (vehicle); ^xx^ *P* < 0.01, compared with B2fc; ^##^ *P* < 0.01, compared with siCCM3.

As detected by RT^2^‐PCR, the mRNA level of *CCM3* was reduced to 23% of the control (Neg.C) after the transfection with siCCM3 (*P* < 0.001), concomitantly accompanied by a 2.3‐fold up‐regulation of EphB4 mRNA (*P* < 0.05) (Fig. 1A). Western blotting confirmed a 35% down‐regulation of CCM3 protein expression (*P* < 0.01), whereas a 3.8‐fold (*P* < 0.01) and a 2.2‐fold (*P* < 0.01) up‐regulation of EphB4 and p‐Erk1/2 (a downstream protein of EphB4 forward signalling), respectively, were detected in the same blot (Fig. 1B). Immunofluorescent staining revealed an enhanced immunoreactivity of EphB4 in *CCM3*‐silenced endothelial cells (Fig. 1C). ELISA detection of p‐EphB4 showed that silencing *CCM3* led to a 66% increase in the level of p‐EphB4 in comparison with the control (*P* < 0.05), mimicking the effect of ephrinB2‐Fc (B2fc) (1 μg/ml), a positive control of EphB4 activation. Furthermore, the activation of EphB4 caused by either CCM3‐silence or by B2fc treatment was completely reversed by the specific EphB4 kinase inhibitor NVP (Fig. 1D).

### Inhibition of EphB4 activity rescued the hyper‐angiogenic phenotype caused by *CCM3*‐ablation *in vitro* and *in vivo*

**Figure 2:**
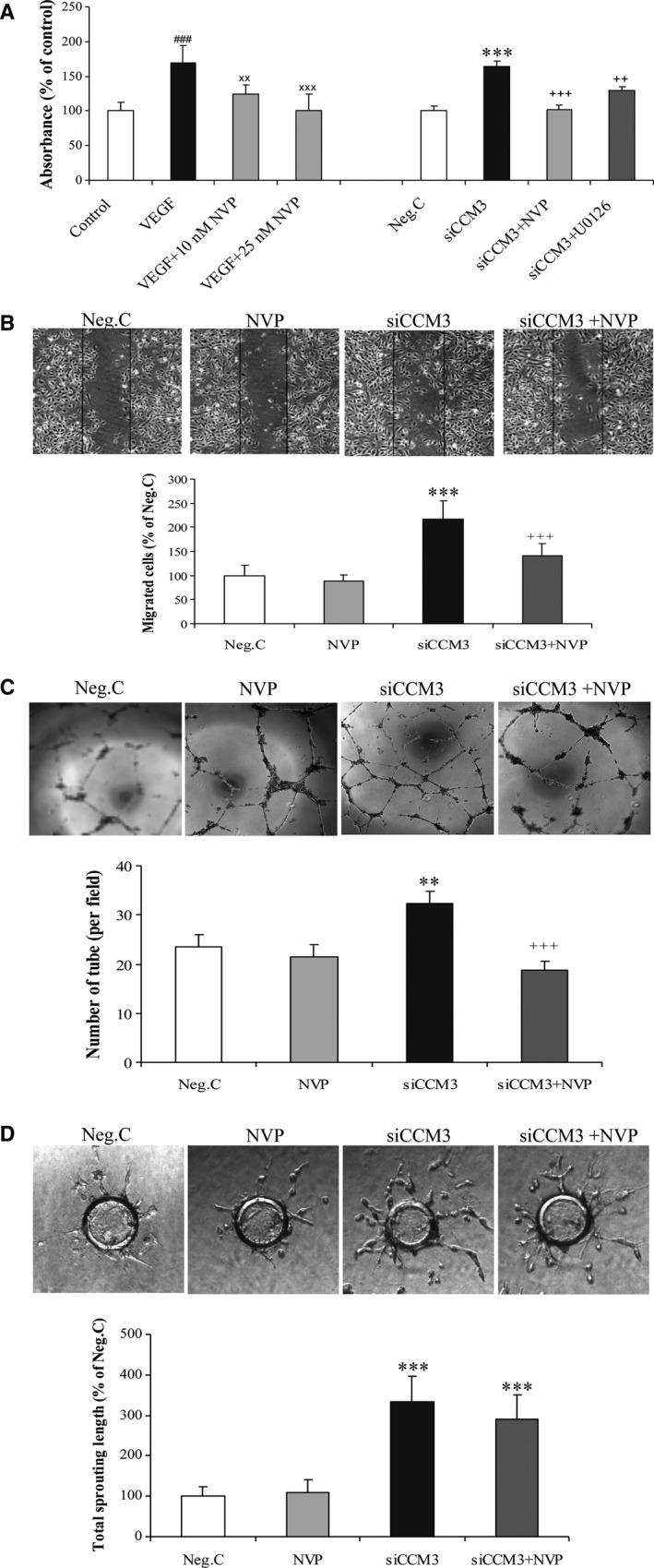
Treatment with EphB4 kinase inhibitor suppressed *CCM3*‐silence‐induced endothelial proliferation (**A**), migration (**B**) and tube formation (**C**), but not sprouting (**D**). As positive control, cells were treated with VEGF (100 ng/ml). For *CCM3‐* or Neg.C‐transfected cells, NVP (10 nM) or U0126 (10 μM) was added to the culture 48 hrs after the transfection. All data presented in A–D were representative of at least three independent experiments. **P < 0.01 and ***P < 0.001, compared with Neg.C; ^###^ *P* < 0.001, compared with control; ^xx^ *P* < 0.01 and *P* < 0.001, compared with VEGF. ^++^ *P* < 0.01 and ^+++^ *P* < 0.001, compared with siCCM3.

Silencing *CCM3* significantly stimulated cell proliferation, which was entirely reversed by the treatment with NVP or with the specific MEK inhibitor U0126 (Fig. 2A). A similar inhibition of the proliferation by NVP was observed in VEGF‐stimulated cells. Silencing *CCM3* also significantly promoted endothelial migration (Fig. 2B), tube formation (Fig. 2C) and sprouting (Fig. 2D), which is in accordance with our previous report 22. Of note, the treatment with NVP abolished siCCM3‐induced increase in cell migration and tube formation, but did not significantly influence endothelial sprouting.

**Figure 3:**
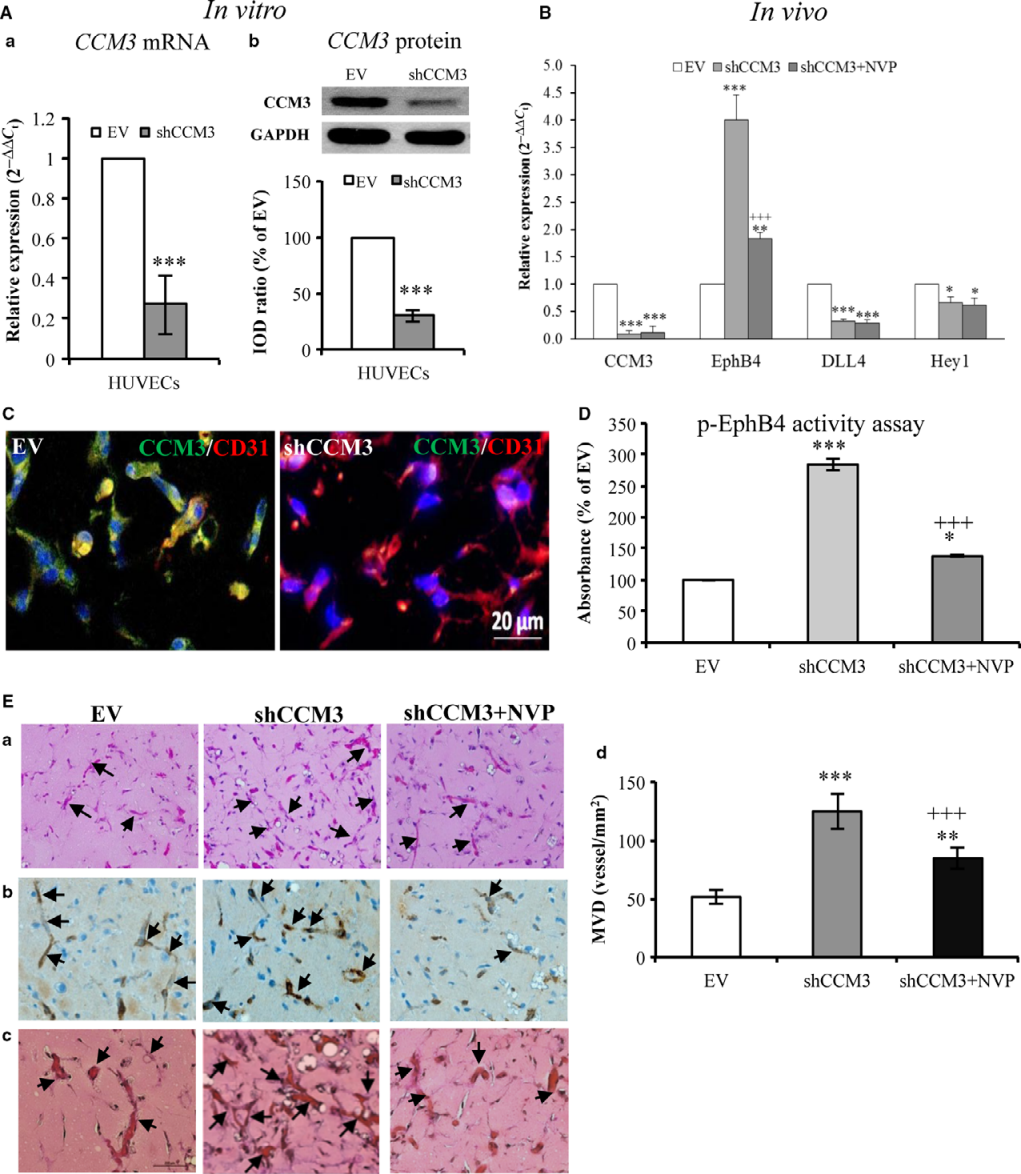
Stable knock‐down of endothelial *CCM3* increased the microvessel density (MVD) in a spheroid‐based angiogenesis model in mice, which was reversed by the treatment with NVP. (**A**) Confirmation of the down‐regulation of CCM3 at mRNA (a) and protein (b) levels before implantation. The expression of mRNA and protein of CCM3 was detected by RT ^2^‐PCR and by Western blot, respectively, after the transduction of shCCM3 or empty vector (EV). (**B**) Down‐regulation of *CCM3* mRNA was associated with the up‐regulation and EphB4 and with the inhibition of *DLL4* and *Hey1 in vivo*. NVP was treated to mice (8 mg/kg, i.g.) every 2nd day. The control mice received vehicle only. The plugs were removed for RT ^2^‐PCR from mice 20 days after the implantation. (**C**) Absence of the CCM3 immunoreactivity in the plugs from shCCM3‐mice. Double staining of CCM3 and CD31 was performed on the sections prepared from plugs. CCM3 was co‐stained with CD31 on the control sections (EV), whereas CCM3 immunoreactivity was missing on the sections from shCCM3‐mice. (**D**) Treatment with NVP significantly inhibited shCCM3‐mediated increase in EphB4 kinase activity in plugs. The level of phosphor‐EphB4 (p‐EphB4) was detected in the protein extracts of plug by ELISA. (**E**) Treatment with NVP reduced shCCM3‐mediated increase in the microvascular density (MVD). H&E staining (a), CD31 immunostaining (b) and elastic Van Gieson staining (EVG) (c) revealed a denser capillary‐like microvasculature in mice implanted with shCCM3‐transduced endothelial cells compared with EV. Quantitative analysis of MVD indicated a significant increase in MVD in the plugs removed from mice implanted with shCCM3‐transduced endothelial cells (shCCM3), which was reduced by the treatment with NVP (d) (shCCM3 + NVP). Arrows in a–c indicate microvessel‐like structure in plug sections. *P ˂ 0.05, **P ˂ 0.01 and ***P ˂ 0.001, compare to EV; ^+++^ *P* ˂ 0.001, compared to shCCM3.

Next, we further confirmed the role of EphB4 in *CCM3*‐silence‐mediated angiogenesis *in vivo*, As shown in Figure 3A, both mRNA (a) and protein (b) levels of endothelial CCM3 were significantly down‐regulated in shCCM3‐endothelial cells compared with the control (EV) before implantation. Down‐regulation of CCM3 was also proven at the mRNA (Fig. 3B) and protein level (Fig. 3C) in the plugs (*in vivo*) taken from mice after 20 days of implantation (*P* < 0.001). Under this knock‐down condition, we detected a significant up‐regulation of EphB4 mRNA (*P* < 0.001) (Fig. 3B) and a marked increase in EphB4 kinase activity in plug (Fig. 3D) (*P* < 0.001). Moreover, the stable knock‐down of endothelial *CCM3* significantly stimulated microvessel formation *in vivo* as revealed by H&E staining (Fig. 3E‐a) and by CD31 immunostaining (Fig. 3E‐b) on the sections prepared from the plugs. Quantitative analysis indicated a 2.5‐fold increase in microvessel density (MVD) in shCCM3 plugs (Fig. 3E‐d). Of note, treatment with NVP not only reversed the elevated levels of mRNA (Fig. 3B) and kinase activity of EphB4 (Fig. 3D), but also inhibited neo‐angiogenesis (Fig. 3E) resulting from *CCM3‐*ablation.

### EphB4 was a downstream kinase of DLL4‐Notch signalling in endothelial cells under the basal condition

**Figure 4:**
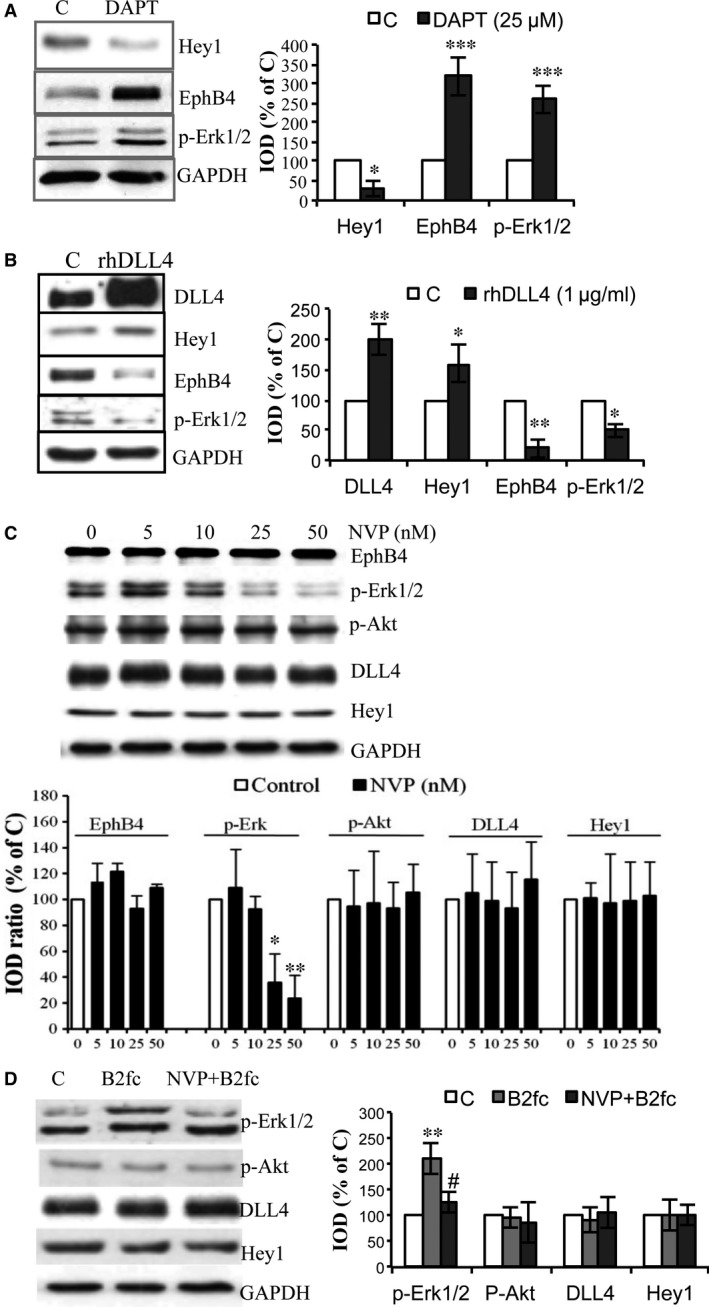
Modulation of the DLL4‐Notch signalling affected the expression of EphB4 and Erk1/2 phosphorylation. (**A**) Inhibition of Notch signalling by DAPT up‐regulated the expression of EphB4 and p‐Erk1/2. Cells were treated with the γ‐secretase inhibitor DAPT (25 μM) for 24 hrs. (**B**) Activation of Notch signalling by rhDLL4 inhibited the expression of EphB4 and p‐Erk1/2. Cells were cultured in dishes pre‐coated with 0.2% gelatin containing 1.0 μg/ml of recombinant human DLL4 (rhDLL4) or the same volume of vehicle (0.1% BSA). *P < 0.05, **P < 0.01 and ***P < 0.001 in A and B, compared with the control. (**C**) The treatment with NVP inhibited the Erk1/2 activation, but did not influence p‐Akt, DLL4 and Hey1 expression. Total protein was extracted 90 min. after treatment with NVP for Western blot. *P < 0.05 and **P < 0.01, compared with control (vehicle). (**D**) Stimulating EphB4 forward signalling by B2fc activated Erk1/2, but did not influence the expression of p‐Akt and DLL4 and Hey1. Cells received NVP treatment (10 nM) for 60 min. followed by the treatment with B2fc (1 μg/ml) for 30 min. **P < 0.01, compared with the control (C); ^#^ *P* < 0.05, compared with B2fc.

We have previously identified DLL4‐Notch signalling targeted by *CCM3‐*silence. Here we further studied whether modification of DLL4‐Notch signalling affected EphB4 forward signalling in endothelial cells. As shown in Figure 4A, the treatment of HUVEC with DAPT inhibited Notch signalling as proved by a significant down‐regulation of Notch target Hey1 (*P* < 0.05). Of note, DAPT treatment also resulted in a three‐fold increase in EphB4 expression (*P* < 0.001) accompanied by a 2.5‐fold up‐regulation of p‐Erk1/2 (*P* < 0.001) (Fig. 4A). In contrast, stimulation of DLL4‐Notch signalling by the treatment with recombinant human DLL4 (rhDLL4) markedly up‐regulated the expression of DLL4 and the target Hey1 and, at the same time, significantly suppressed the expression of EphB4 and p‐Erk1/2 (Fig. 4B). These results indicate that DLL4‐Notch signalling acts upstream of EphB4 and regulates the expression of EphB4 in endothelial cells.

Next, we examined whether modulation of EphB4 activity affected DLL4‐Notch signalling. Interestingly, neither inhibition nor activation of EphB4, respectively, by NVP (Fig. 4C) or by B2fc (Fig. 4D) affected the expression of DLL4 and Hey1, confirming that DLL4‐Notch signalling relies upstream of EphB4 forward signalling under the basal condition. As shown in Figure 4C, activation of p‐Erk1/2 was observed in control HUVEC, which is consistent with our previous finding 21, 22. Treatment with NVP induced a dose‐dependent inhibition of p‐Erk1/2 without affecting EphB4 protein expression (Fig. 4C), whereas B2fc led to an up‐regulation of p‐Erk1/2, which was reversed by NVP. These data indicate that Erk1/2 is a target of EphB4 forward kinase signalling.

### *CCM3*‐silence‐resulted activation of EphB4 in endothelial cells was mediated by DLL4‐Notch signalling

**Figure 5:**
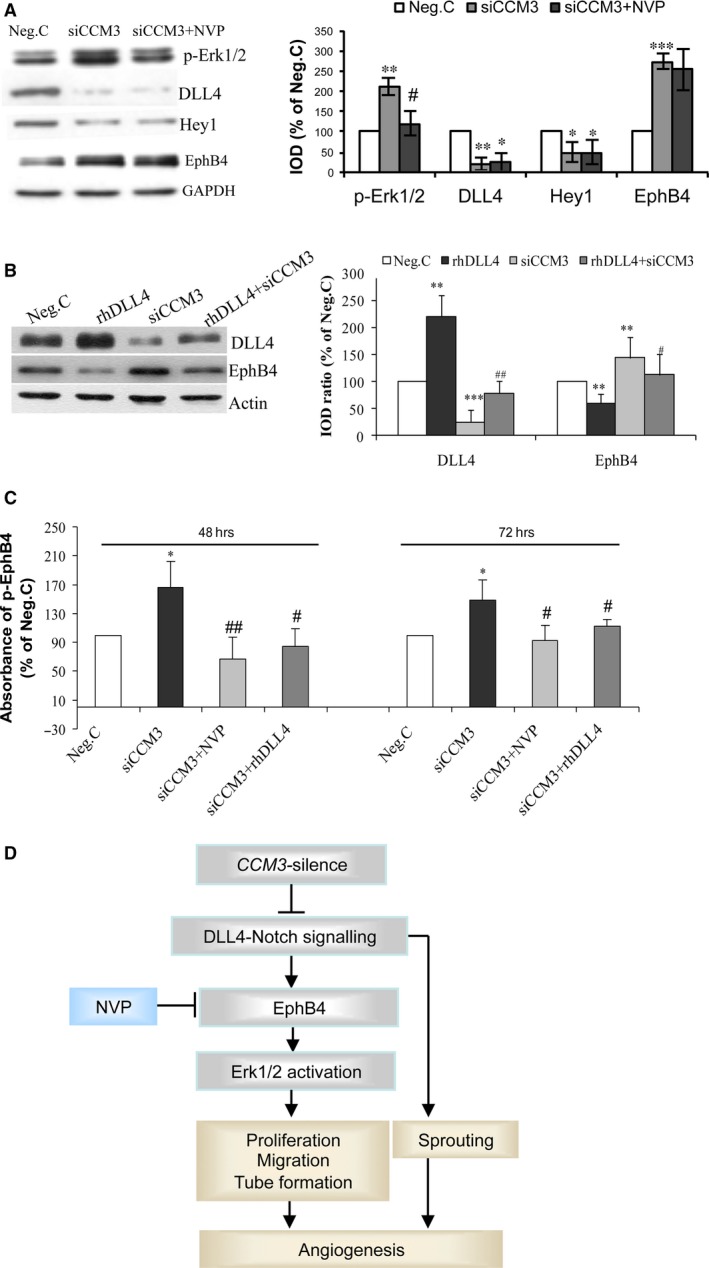
Crosstalk of EphB4 forward signalling and DLL4‐Notch signalling after *CCM3‐*silence. (**A**) *CCM3‐*silence‐induced suppression of DLL4‐Notch signalling was not affected by NVP. Total protein was extracted from the cells treated with NVP (10 nM) or with vehicle for 90 min for Western blot. *P < 0.05, **P < 0.01 and ***P < 0.001, compared with Neg.C; ^#^ *P* < 0.05, compared with siCCM3. (**B**) *CCM3‐*silence‐mediated up‐regulation of EphB4 protein was reversed by the treatment with rhDLL4. The total protein was extracted from HUVECs 72 hrs after transfection with Neg.C or with siCCM3 in the presence or the absence of rhDLL4 (1 μg/ml) for Western blot analysis. *P < 0.05, **P < 0.01 and ***P < 0.001, compared with Neg. C; ^#^ *P* < 0.05, ^##^ *P* < 0.01, compared with siCCM3. (**C**) *CCM3‐*silence‐mediated activation of EphB4 was reversed by rhDLL4 and by NVP. The level of p‐EphB4, reflecting the activity of EphB4, was detected by ELISA. *P < 0.05, compared with Neg.C; ^#^ *P* < 0.05 and ^##^ *P* < 0.01, compared with siCCM3. (**D**) Schematic illustration of the signalling pathways affected by CCM3‐ablation.

Consistent with our previous finding 22, *CCM3*‐silence significantly down‐regulated the expression of DLL4 and its target Hey1, which was not reversed by treatment with NVP (Fig. 5A), indicating *CCM3*‐silence‐mediated Notch inhibition is not influenced by EphB4 kinase inhibition. On the other hand, treatment of cells with rhDLL4 significantly reversed not only siCCM3‐mediated down‐regulation of DLL4, but also siCCM3‐mediated up‐regulation of EphB4. Moreover, treatment of cells with either NVP or rhDLL4 abolished siCCM3‐induced increase in p‐EphB4 (Fig. 5C). These data demonstrate an endothelial signalling pathway of DLL4‐Notch‐EphB4 forward signalling underlying *CCM3*‐ablation.

## Discussion

CCM3 is a ubiquitous protein expressed in nearly all tissues and in various types of cells including endothelial cells, neuronal cells and glial cells. Deletion of *ccm3* leads to mouse embryonic lethality due to vascular defects. Loss‐of‐function mutation of *CCM3* causes CCM involving aberrant angiogenesis 1. Knock‐down of endothelial *CCM3* largely stimulates tumour angiogenesis and promotes GBM tumour growth 23. Thus, further characterization of the signalling underlying CCM3‐ablation‐mediated angiogenesis may not only extend our fundamental understanding of this protein, but also help to reveal the crucial role of CCM3 in diseases. The present study provides evidence that CCM3‐ablation targets EphB4 forward signalling. This is supported by the data showing a significant up‐regulation of EphB4 expression, an increased level of p‐EphB4 and a concomitant activation of Erk1/2 after silencing *CCM3*; moreover, treatment with a specific EphB4 kinase inhibitor reversed *CCM3*‐silence‐mediated activation of EphB4 forward signalling and rescued the hyper‐angiogenic phenotype induced by siCCM3 *in vitro* and *in vivo*. Our study further identified a signalling cascade of DLL4‐Notch‐EphB4‐Erk1/2 targeted by CCM3‐ablation. These findings indicate EphB4 as a key modulator, besides DLL4‐Notch, in the angiogenesis stimulated by *CCM3*‐deficiency.

EphB4 is preferentially expressed by venous endothelial cells. Binding of EphB4 with its ligand ephrinB2 induces bi‐directional signalling and regulates diverse endothelial functions in development and in diseases. Upon engagement of EphB4 with its ligand, EphB4 becomes tyrosine phosphorylated through autophosphorylation on its kinase domain, thereby activating kinase‐dependent forward signalling, whereas the reverse signalling is activated upon ephrinB2 tyrosine phosphorylation through recruitment of itself 25. Martiny‐Baron *et al*. 26 showed a specific kinase inhibitor of the EphB4, NVP (NVP‐BHG712), that inhibited EphB4 autophosphorylation, thereby suppressing EphB4 forward signalling *in vitro* and *in vivo*. Our *in vitro* study demonstrated that NVP induced a dose‐dependent (5–50 nM) inhibition of p‐Erk1/2 without changing EphB4 protein expression under the basal condition (Fig. 4C); moreover, NVP at the concentration of 10 nM significantly inhibited the B2Fc‐ (Fig. 1D) and *CCM3*‐silence‐mediated activation of EphB4 (Fig. 5C), but did not alter EphB4 protein expression. These data identify NVP as a kinase inhibitor affecting EphB4 forward signalling. Importantly, the hyper‐angiogenic phenotype induced by *CCM3‐*silence *in vitro* (Fig. 2) was reversed by NVP treatment, pointing out a crucial role of EphB4 forward signalling in *CCM3*‐ablation‐mediated angiogenesis. The data derived from *in vivo* experiments further demonstrated that NVP sufficiently inhibited EphB4 kinase activity (Fig. 3D) and rescued the neo‐angiogenesis induced by *CCM3*‐silence (Fig. 3E). Interestingly, NVP also unexpectedly inhibited EphB4 mRNA expression *in vivo* (Fig. 3B). This phenomenon could be caused by a higher plasma level of NVP under this treatment condition (8 mg/kg, i.g., every 2nd day), in spite of using a lower dosage and frequency of NVP treatment in the present study compared with that used in the study by Martiny‐Baron *et al*. 26. Nevertheless, treatment dosage with NVP in animals needs to be further optimized in the future, although the suppression of EphB4 mRNA by NVP does not induce the opposite effect, and could rather additively contribute to its inhibition of EphB4 kinase activity.

DLL4‐Notch is another pivotal signalling pathway for regulating vascularisation, angiogenesis as well as post‐angiogenic vessel remodelling and maturation 14. We have previously demonstrated that loss of CCM3 stimulated endothelial angiogenesis via inhibition of DLL4‐Notch signalling 22. Thus, it is of particular interest to find out how these two identified signalling pathways interplay with each other in regulating endothelial angiogenesis. Here, we showed that inhibiting DLL4‐Notch signalling by γ‐secretase inhibitor DAPT increased EphB4 expression. In contrast, activation of DLL4‐Notch signalling by rhDLL4 down‐regulates the EphB4 protein expression. Furthermore, treatment with rhDLL4 inhibited EphB4 kinase activity in *CCM3*‐silenced endothelial cells (Fig. 5C). On the other hand, activation or inhibition of EphB4 kinase activity by B2fc or NVP, respectively, did not alter DLL4‐Notch signalling (Fig. 4C and D). *In vivo* study further confirmed that inhibition of EphB4 by NVP did not alter the expression of *DLL4* and *Hey1*,* a* target gene of DLL4‐Notch signalling (Fig. 3B). These results indicate that DLL4‐Notch signalling acts upstream of EphB4 and negatively regulates the expression and activity of EphB4 in endothelial cells under basal condition as well as after silencing *CCM3*. Of note, the blockage of *CCM3‐*silence‐induced massive endothelial sprouting by rhDLL4 22 but not by NVP (Fig 2D) indicates that *CCM3‐*ablation‐induced sprouting is mediated by inhibition of DLL4‐Notch signalling. These data suggest that DLL4‐Notch and EphB4 signalling could also act individually, apart from their function in the coordinated cascade. In addition, the present study demonstrated that Erk1/2 is at least one important target of the CCM3‐DLL4‐Notch‐EphB4 signalling cascade.

In conclusion, we defined EphB4 as a novel mediator of the angiogenesis resulted from *CCM3*‐ablation. Loss of CCM3 leads to the up‐regulation and activation of EphB4 via inhibiting DLL4‐Notch signalling, resulting in subsequent activation of Erk1/2 and eventual stimulation of angiogenesis (Fig. 5D). Identification of the endothelial signalling pathway of CCM3‐DLL4‐Notch‐EphB4‐Erk1/2 constitutes the mechanism of *CCM3*‐deficiency‐mediated angiogenesis and thus may potentially contribute to new therapeutic concepts in disrupting aberrant angiogenesis in human diseases such as CCM and hyper‐vascularized tumours.

## Conflict of interest

The authors confirm that there are no conflicts of interest.
